# A Novel 4H-SiC MESFET with a Heavily Doped Region, a Lightly Doped Region and an Insulated Region

**DOI:** 10.3390/mi12050488

**Published:** 2021-04-26

**Authors:** Hujun Jia, Mengyu Dong, Xiaowei Wang, Shunwei Zhu, Yintang Yang

**Affiliations:** School of Microelectronics, Xidian University, Xi’an 710071, China; mengyudong@stu.xidian.edu.cn (M.D.); boywangxw@126.com (X.W.); swzhu@stu.xidian.edu.cn (S.Z.); ytyang@xidian.edu.cn (Y.Y.)

**Keywords:** SiC, Metal-Semiconductor Field Effect Transistor (MESFET), heavily doped region, power added efficiency (PAE)

## Abstract

A novel 4H-SiC MESFET was presented, and its direct current (DC), alternating current (AC) characteristics and power added efficiency (PAE) were studied. The novel structure improves the saturation current (I_dsat_) and transconductance (g_m_) by adding a heavily doped region, reduces the gate-source capacitance (C_gs_) by adding a lightly doped region and improves the breakdown voltage (V_b_) by embedding an insulated region (Si_3_N_4_). Compared to the double-recessed (DR) structure, the saturation current, the transconductance, the breakdown voltage, the maximum oscillation frequency (f_max_), the maximum power added efficiency and the maximum theoretical output power density (P_max_) of the novel structure is increased by 24%, 21%, 9%, 11%, 14% and 34%, respectively. Therefore, the novel structure has excellent performance and has a broader application prospect than the double recessed structure.

## 1. Introduction

The third-generation semiconductor is the development trend of the semiconductor. The third-generation semiconductor material is mainly divided into silicon carbide and gallium nitride. Silicon carbide is more suitable as a substrate material. Compared to the first- and second-generation semiconductors, it has a higher breakdown voltage, wider band gap, higher electrical conductivity and thermal conductivity, and will replace the previous two generations of semiconductor materials in some fields, such as high temperature, high pressure, high power, high frequency, etc. [1,2,3]. Due to the lack of large-size single crystals of gallium nitride, the main forms of the third-generation semiconductor materials are silicon carbide-based silicon carbide epitaxial devices, silicon carbide-based gallium nitride epitaxial devices, silicon carbide is more widely used [4]. Benefiting from the advantages of silicon carbide materials, silicon carbide-based MESFETs have better performance than silicon-based and gallium arsenide-based devices [5].

In addition, since the depletion layer isolates the carriers from the surface of the device during the operation of the MESFETs, the surface of the device has a relatively small effect on the carriers. Therefore, compared with the MOSFETs, the MESFETs have higher saturated electron mobility, higher current, greater transconductance and transmission frequency [6]. Therefore, the application of 4H-SiC MESFET in the fields of high temperature, high voltage, high frequency and high power has received extensive attention. How to improve the performance of 4H-SiC MESFET has become a challenge. Methods such as adding field plates [7], improving gate structure [8,9,10] and improving channel structure [11,12,13,14] have been used to improve the performance of devices. With the continuous reduction in device size, the requirements of device design for power consumption and efficiency become increasingly higher. In recent years, how to improve the PAE of devices without significantly sacrificing DC and AC characteristics has gradually become an important topic [15,16,17].

In this work, we propose a novel 4H-SiC MESFET with a heavily doped region, a lightly doped region and an insulated region. Due to the existence of a lightly doped region, heavily doped region and insulated region, the I_dsat_, V_b_, P_max_ and f_max_ of the novel structure are greater than those of the DR structure [18]. We also explore the maximum PAE of the devices.

## 2. Device Structure and Simulation Methods

**Figure 1:**
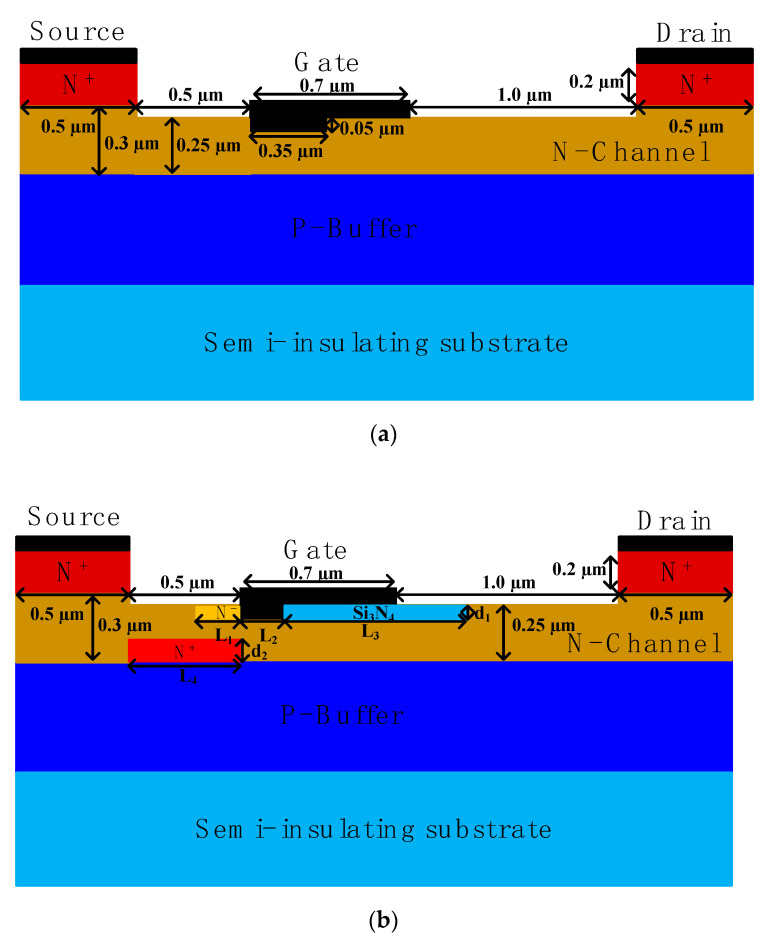
Cross-sectional diagrams of (**a**) the DR structure and (**b**) the proposed structure.

Figure 1 shows the cross-sectional views of the DR structure and the novel structure. Compared to traditional DR structure, in addition to the substrate, buffer and channel layer, the novel structure also has an additional heavily doped region, a lightly doped region and an insulated region in the channel. Table 1 shows the structure parameters of the devices.

The novel structure can be made using similar processes to those reported in [19]. The heavily doped region and lightly doped region can be formed by ion implantation and activation processes. The fabrication of the insulated region can refer to the process steps described in [10]. First, the location of the metal gate and insulating area is created by etching; then, the oxide layer is grown on the surface and the silicon nitride is etched and deposited by using a mask, and finally, the metal gate is manufactured by a similar method.

**Figure 2:**
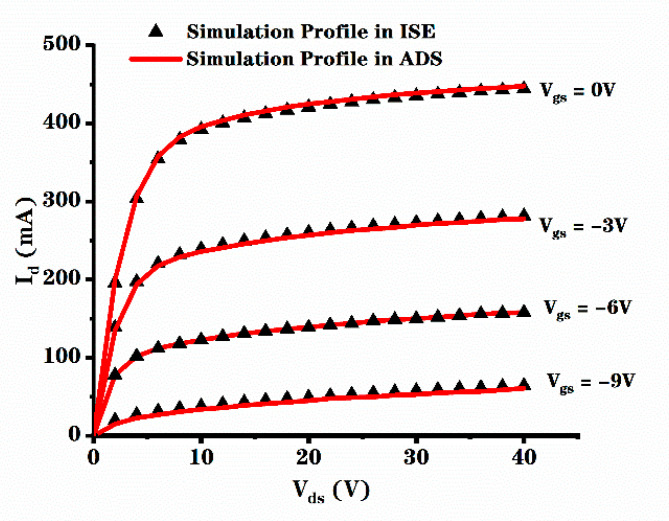
Comparison of simulation data in ISE and ADS on output current.

Two-dimensional numerical simulation of these structures was carried out by ISE-TCAD software. As the process of SiC material in ADS software is not perfect at present, while the process of GaAs material is mature, we fit the model of SiC MESFET by modifying the model parameters of GaAs MESFET, so that it could better reflect the trend of power added efficiency of devices. After the device model is established, it needs to be verified, and the “ads_templates: FET_curve_tracer” module in ADS was used to measure its IV characteristics; the measurement results were compared with those in ISE-TCAD. The results are shown in Figure 2. It can be seen that the IV characteristic curve of devices in ADS is in good agreement with that in ISE-TCAD. Therefore, we used the EE_FET3 model of nonlinear GaAsFET model in ADS, modified the existing model according to the data obtained from simulation and used “Load Pull-PAE, Output Power Contours” in Power Amplifier Examples to measure the power added efficiency [15].

## 3. Simulation Results and Discussion

**Figure 3:**
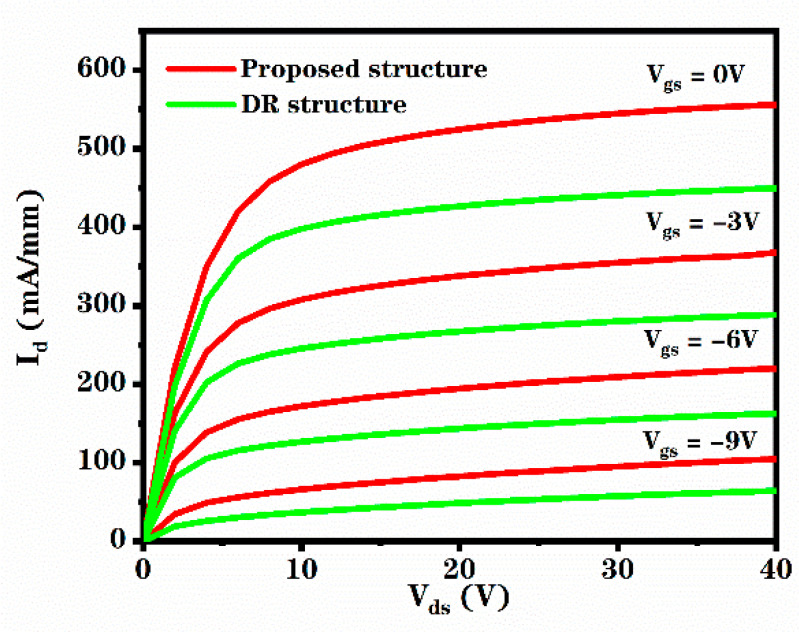
Evolution of drain current (I_d_)as a function of drain-source voltage (V_ds_) for the proposed structure and DR structure.

Figure 3 shows the I-V characteristics of the novel structure and the DR structure. The gate bias voltages (V_gs)_ of these curves in the figure are −9 V, −6 V, −3 V and 0 V, respectively. It can be calculated that the I_dsat_ of the novel structure is about 24% greater than the I_dsat_ of the DR structure, due to the existence of heavily doped region. The heavily doped region provides more carriers for the device to have a larger saturation current, which means greater output power and better output characteristics.

**Figure 4:**
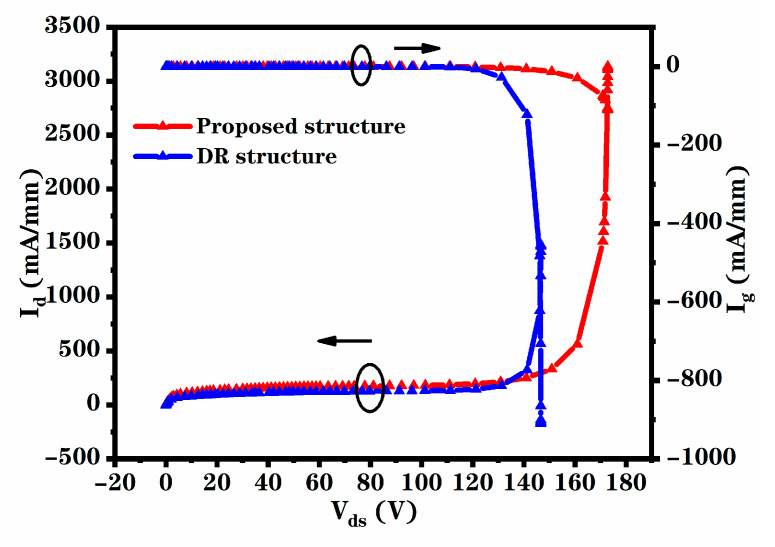
Breakdown performance of the proposed structure and DR structure.

**Figure 5:**
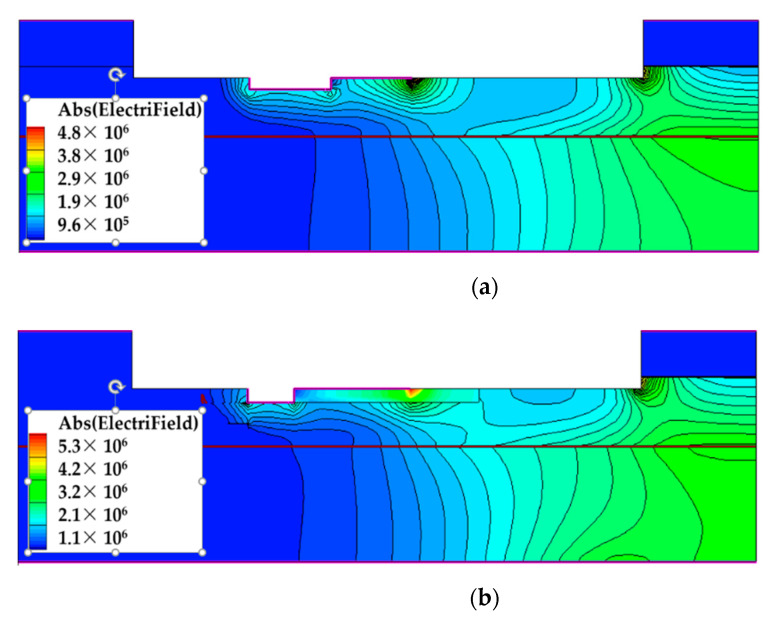
Potential distribution of the (**a**) DR structure and (**b**) proposed structure.

Breakdown voltage is an important parameter for MESFET devices, which limits P_max_ and applications of the devices. Figure 4 shows the breakdown performance of the two structures under the condition of V_gs_ = V_t_. The variation of drain current and gate current (I_g_) with the increase in V_ds_ is shown in the figure. Studies show that breakdown is caused by the accumulation of the electric field of the device [20]. With the increase in V_ds_, the maximum electric field increases continuously, which makes the carriers accelerate and collide with each other seriously. Finally, the device is broken down due to the large increase in carriers [10]. It can be found that the breakdown performance of the novel structure is better in contrast with the DR structure, because the insulation region improves the electric field distribution in the device, as shown in Figure 5.

The output power density was greatly improved due to the increase in I_dsat_ and Vb. The calculation expression for its maximum theoretical output power density is as follows [21]:

(1) $$P_{\max}=\frac{I_{\mathrm{dsat}}\left(V_{b}-V_{\mathrm{keen}}\right)}{8}$$

where I_dsat_ is the saturated drain current under the conditions of V_gs_ = 0 V and V_ds_ = 40 V, and V_knee_ is the knee voltage. The P_max_ of the DR structure is 5.8 W/mm and the P_max_ of the novel structure is 7.8 W/mm. It can be seen that the P_max_ is increased by about 34%.

**Figure 6:**
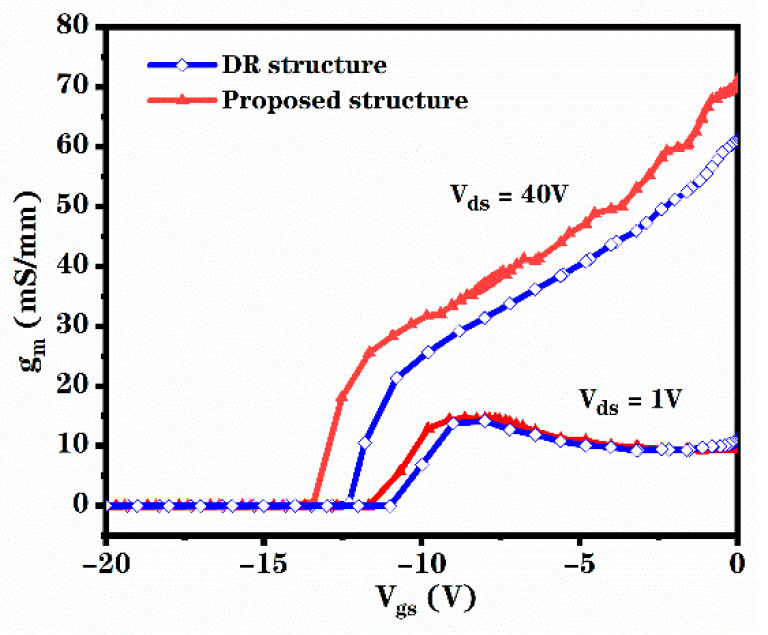
Relationship between gm and V_gs_ for the proposed structure and DR structure at V_ds_ = 40 V and V_ds_ = 1V.

DC transconductance reflects the relationship between gate voltage and drain-source current. The larger the DC transconductance, the stronger the gate control capability. The relationship between the DC transconductance of these two structures and the gate voltage at V_ds_ = 40 V and V_ds_ = 1 V is shown in Figure 6. As shown in the figure, the DC transconductance of the novel structure is greater than the DC transconductance of the DR structure. Compared to the DR structure, the DC transconductance of the novel structure is increased by 21%.

**Figure 7:**
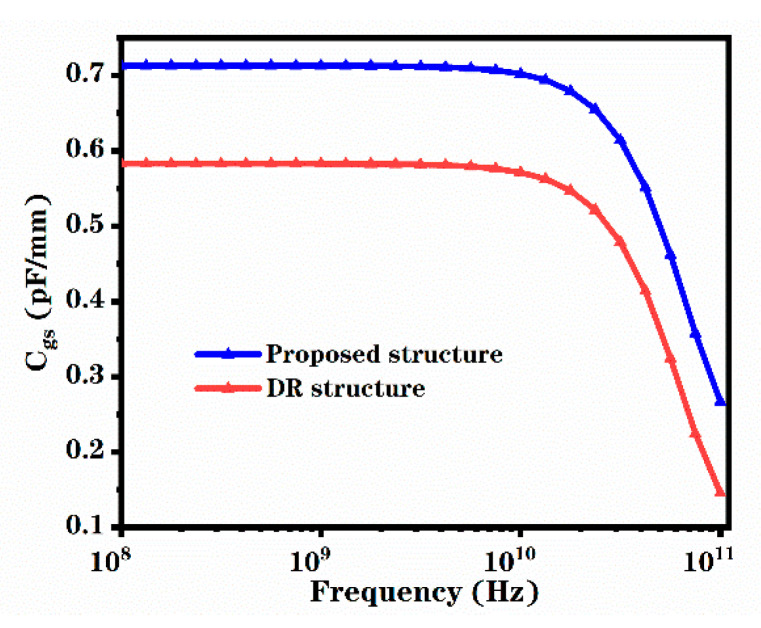
Relationship between C_gs_ and frequency for the proposed structure and DR structure at V_gs_ = 0 V and V_ds_ = 40 V.

The gate-source capacitance (C_gs_) is a significant parameter closely related to the AC characteristics. Additionally, the reduction in the C_gs_ can increase the f_T_ of the device. Under the condition of V_ds_ = 40 V and V_gs_ = 0 V, the relationship between C_gs_ and frequency of DR structure and novel structure is shown in Figure 7. As can be seen, the C_gs_ of the novel structure increases by 22% compared to the DR structure. The cut-off frequency is given by the following formula [22]:

(2) $$f_{T}=\frac{g_{m}}{2{\pi C}_{\mathrm{gs}}}$$

where *g*_m_ is the DC transconductance. Although the increase in C_gs_ will decrease the f_T_, the increase of DC transconductance will increase the f_T_. Finally, the f_T_ of the novel structure is only reduced by 1% compared to the DR structure.

**Figure 8:**
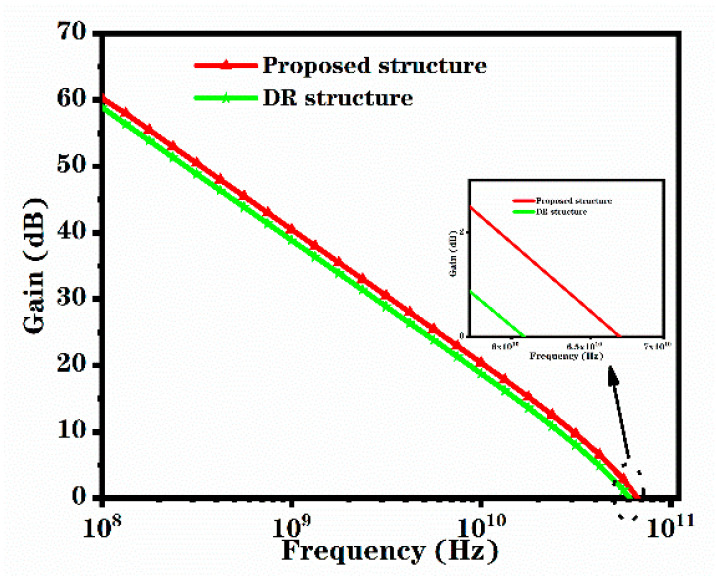
Relationship between unilateral power gain and frequency for the proposed structure and DR structure at V_gs_ = 0 V and V_ds_ = 40 V.

Figure 8 shows the relationship between the simulated unilateral power gain and frequency of the two structures under the conditions of V_gs_ = 0 V and V_ds_ = 40 V. The maximum oscillation frequency is considered to be the frequency when the gain is zero. The f_max_ of the novel structure is 66.9 GHz, while the f_max_ of the DR structure is 60.5GHz. The formula for calculating the maximum oscillation frequency is as follows [22]:

(3) $$f_{\max}=\frac{f_{T}}{2}\sqrt{\frac{R_{\mathrm{ds}}}{R_{g}}}$$

where R_ds_ is the drain-source resistance and R_g_ is the gate resistance. As the novel structure has a heavily doped region, the R_ds_ and R_g_ of the novel structure are smaller than those of the DR structure, but the decrease proportion of the R_g_ is larger than that of the R_ds_, so the f_max_ is increased. Therefore, the novel structure has better RF characteristics.

**Figure 9:**
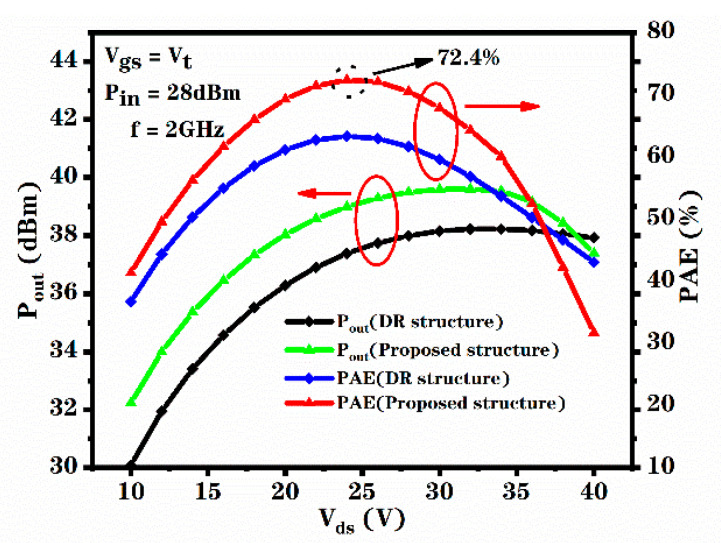
Relationship between PAE and V_ds_ for the DR structure and proposed structure.

**Figure 10:**
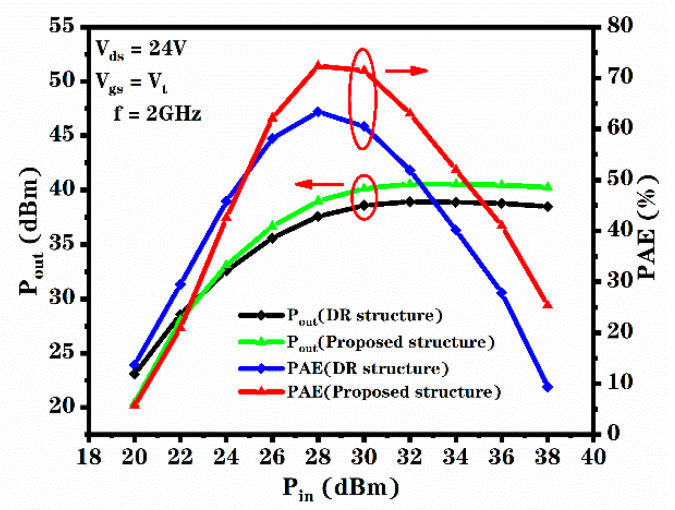
Relationship between PAE and P_in_ for the DR structure and proposed structure.

Power added efficiency is an important indicator in device design in recent years. Larger power added efficiency is more conducive to improving the power consumption of devices and is more in line with the concept of energy conservation and emission reduction. Power added efficiency is the increase in RF power produced by the amplifier, divided by the total DC input power [23]:

(4) $$\mathrm{PAE}=100\frac{\left(P_{\mathrm{out}}-P_{\mathrm{in}}\right)}{P_{\mathrm{dc}}}$$

where Pout is alternating current output power, Pin is alternating current input power and P_dc_ is direct current input power. The PAE versus the V_ds_ for the novel structure and DR structure is shown in Figure 9. It can be seen that the PAE curve of the novel structure is mostly above the PAE curve of the DR structure. Additionally, the PAE versus the RF input power for the DR structure and novel structure is shown in Figure 9. It can be seen that with the increase in the amplitude of the input signal, the amplifier enters the saturation state, and the output power begins to saturate, while the PAE and gain decrease. Therefore, with the increase in input power, the output power is finally saturated, which reduces the gain and the PAE [23]. From Figure 9 and Figure 10, we calculated that the maximum PAE of the novel structure increased by 14% to 72.4% due to the increase in transconductance, compared to the DR structure. As the transconductance of the device increases, the AC gain of the device, the AC output voltage and the AC output current of the device increase, the AC output power of the device increases. In addition, the increase in transconductance makes the DC working current of the device increase and the DC input power of the device increase. However, the influence of increasing transconductance on the AC output power is higher than that on the DC input power. Therefore, the increase in the transconductance of the device increases the PAE of the device.

## 4. Conclusions

A novel 4H-SiC MESFET with a heavily doped region, a lightly doped region and an insulated region was presented, simulated and compared to the conventional DR structure. The DC and AC characteristics and PAE of the two structures were analyzed. The results show that the g_m_ of the novel structure is increased by 21%, the P_max_ of the novel structure is increased by 34% and the maximum PAE of the novel structure is increased by 14%, compared to those of the DR structure. Therefore, the device has better performance and has a more promising application in the microwave field. It is very suitable for use in radar transmitters. The reasonable application of this device can significantly increase the output power and power density of the radar transmitter, increase the working frequency and working frequency bandwidth.

## Figures and Tables

**Table 1:** Structure parameters of the devices.

Parameter	Value
Thickness of source/drain	0.2 μm
Length of source/drain	0.5 μm
Doping of source/drain	1 × 10^20^ cm^−3^
Doping of channel	3 × 10^17^ cm^−3^
Thickness of buffer	0.5 μm
Doping of buffer	1.4 × 10^15^ cm^−3^
Doping of lightly doped region	1 × 10^15^ cm^−3^
Doping of heavily doped region	5 × 10^19^ cm^−3^
L_1_	0.2 μm
L_2_	0.2 μm
L_3_	0.8 μm
L_4_	0.5 μm
d_1_	0.06 μm
d_2_	0.1 μm
